# Supplementary figures and images for: SafeNET: Initial development and validation of a real-time tool for predicting mortality risk at the time of hospital transfer to a higher level of care
Source: PLoS One. 2021 Feb 8;16(2):e0246669. doi: 10.1371/journal.pone.0246669 (PMC7870086; doi:10.1371/journal.pone.0246669)

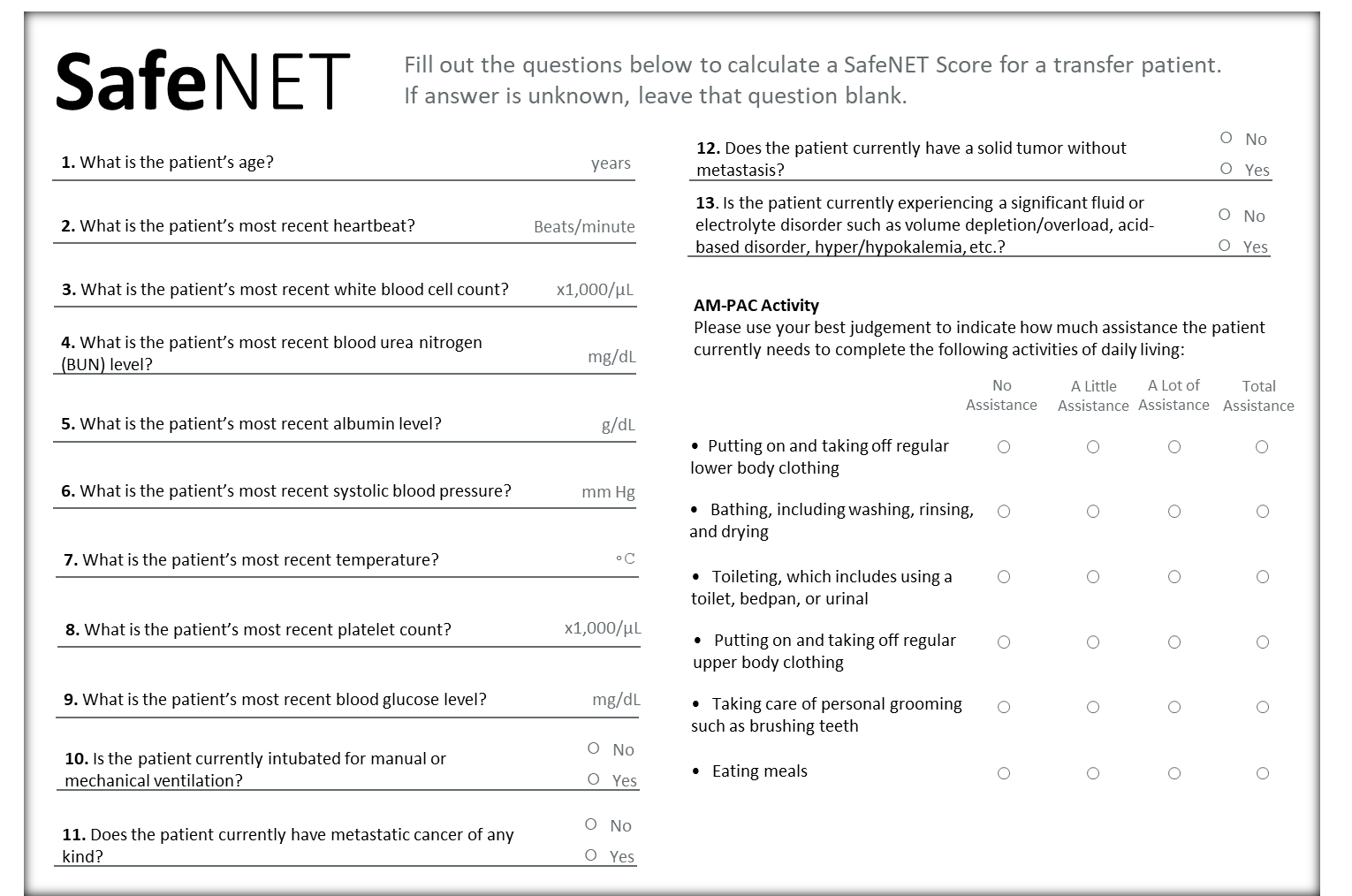
**S1 Fig**. **Web-based SafeNET survey completed at patients’ bedsides.**

Supplement: S1 Fig — (DOCX) [file pone.0246669.s001.docx]
