# Supplementary material for: SafeNET: Initial development and validation of a real-time tool for predicting mortality risk at the time of hospital transfer to a higher level of care
Source: PLoS One. 2021 Feb 8;16(2):e0246669. doi: 10.1371/journal.pone.0246669 (PMC7870086; doi:10.1371/journal.pone.0246669)
Supplement: S1 Table — (DOCX) [file pone.0246669.s002.docx]

**S1 Table. List of 70 patient variables considered during the development of SafeNET.**

| ***Patient Demographics*** | ***Patient Comorbidities^#^*** |
| --- | --- |
| • Age* [11,14,16] | • AIDS/HIV* |
| • Race (Black)* | • Alcohol Abuse* |
| • Race (Other)* | • Blood Loss Anemia* |
| • Sex* [11] | • Cardiac Arrythmias* |
| ***Patient Vital Signs*** | • Chronic Pulmonary Disease* |
| • Diastolic Blood Pressure* [11] | • Coagulopathy* |
| • Heart Rate* [11–16] | • Congestive Heart Failure* [11] |
| • Mean Arterial Pressure* [10,16] | • Deficiency Anemia* |
| • Oxygen % (FiO2) [10] | • Depression* |
| • Oxygen L/min | • Diabetes, Complicated* [11,14] |
| • Oxygen Saturation* [11–16] | • Diabetes, Uncomplicated* [11,14] |
| • Respiratory Rate* [11–17] | • Drug Abuse* |
| • Systolic Blood Pressure* [11–13,15,17] | • Fluid and Electrolyte Disorders* |
| • Temperature (°C)* [11–14,16] | • Hypertension, Complicated* |
| ***Lab Tests*** | • Hypertension, Uncomplicated* |
| • Albumin* [11] | • Hypothyroidism* |
| • Blood Urea Nitrogen* [11] | • Liver Disease* |
| • Creatinine* [10,11] | • Lymphoma* [11] |
| • Glucose* [11] | • Metastatic Cancer* [11] |
| • Hemoglobin* [11] | • Obesity* |
| • Platelets* [10,11] | • Other Neurological Disorders* |
| • White Blood Cells* [11] | • Paralysis* |
| • White Blood Cells/Granulocyte | • Peptic Ulcer Disease* |
| ***Functional/Mental Status*** | • Peripheral Vascular Disorders* |
| • AM-PAC Activity Score* | • Psychoses* |
| • AM-PAC Mobility Score* | • Pulmonary Circulation Disorders* |
| • Glasgow Coma Score [10,11,16,17] | • Renal Failure* [11] |
| ***Respiratory Therapy*** | • Rheumatoid Arthritis* |
| • Mechanical Ventilation Status (Initial, Subsequent) | • Solid Tumor without Metastasis* [11] |
| • Native Airway (Endotracheal, Tracheal) | • Valvular Disease* |
| • Respiratory Device: Mechanical Ventilation* [11] | • Weight Loss* |
| • Respiratory Device: Mild O_2_ Support* [12,13] | ***Code Status*** |
| • Respiratory Device: Moderate O_2_ Support* [12,13] | • CPR Yes: Interventions Limited [11] |
| ***Blood Product Therapy*** | • CPR Yes: No Limitations [11] |
| • Blood Products Administered | • CPR: No |
| • Cryoprecipitate | • Chest Compressions |
| • Other Blood Products | • Comfort Measures Only |
| • Packed Red Blood Cells |  |
| • Plasma |  |
| *Denotes a patient variable included in the 54-variable model because it was either (a) reliably available (≥50% of patients with recorded values) within 3 hours of admission to the receiving hospital or (b) an Elixhauser Comorbidity condition.  ^#^Elixhauser comorbidities defined by ICD-10 codes[20].  Note: References identify previously developed tools that incorporated similar predictors. | |
