# Supplementary material for: SafeNET: Initial development and validation of a real-time tool for predicting mortality risk at the time of hospital transfer to a higher level of care
Source: PLoS One. 2021 Feb 8;16(2):e0246669. doi: 10.1371/journal.pone.0246669 (PMC7870086; doi:10.1371/journal.pone.0246669)
Supplement: S2 Table — (DOCX) [file pone.0246669.s003.docx]

**S2 Table.** **Rank of variable importance of the 54 variables.**

| **Variable** | **In-Hospital Mortality** | **30-Day Mortality** | **90-Day Mortality** | **CMO/Hospice Discharge** | **Average Rank** |
| --- | --- | --- | --- | --- | --- |
|  |  |  |  |  |  |
| Age | 3 | 1 | 1 | 1 | 1.50 |
| AM-PAC Activity Score | 1 | 2 | 2 | 2 | 1.75 |
| Blood Urea Nitrogen | 4 | 3 | 3 | 4 | 3.50 |
| AM-PAC Mobility Score | 6 | 4 | 5 | 3 | 4.50 |
| Fluid and Electrolyte Disorders | 7 | 7 | 6 | 6 | 6.50 |
| Temperature | 5 | 5 | 9 | 11 | 7.50 |
| Respiratory Device: Mechanical Ventilation | 2 | 9 | 14 | 5 | 7.50 |
| Albumin | 15 | 6 | 7 | 7 | 8.75 |
| Glucose | 8 | 8 | 11 | 9 | 9.00 |
| Heart Rate | 12 | 12 | 13 | 10 | 11.75 |
| Systolic Blood Pressure | 11 | 15 | 10 | 12 | 12.00 |
| Platelets | 14 | 11 | 12 | 13 | 12.50 |
| White Blood Cells | 13 | 14 | 16 | 8 | 12.75 |
| Oxygen Saturation | 10 | 13 | 17 | 15 | 13.75 |
| Creatinine | 9 | 16 | 15 | 16 | 14.00 |
| Metastatic Cancer | 29 | 10 | 4 | 14 | 14.25 |
| Mean Arterial Pressure | 17 | 17 | 18 | 17 | 17.25 |
| Respiratory Rate | 16 | 19 | 20 | 18 | 18.25 |
| Solid Tumor w/o Metastases | 30 | 18 | 8 | 20 | 19.00 |
| Diastolic Blood Pressure | 18 | 20 | 21 | 22 | 20.25 |
| Hemoglobin | 21 | 21 | 19 | 21 | 20.50 |
| Other Neurological Disorders | 20 | 24 | 24 | 19 | 21.75 |
| Liver Disease | 22 | 22 | 23 | 23 | 22.50 |
| Respiratory Device: Moderate O_2_ Support | 19 | 25 | 26 | 25 | 23.75 |
| Congestive Heart Failure | 27 | 23 | 22 | 30 | 25.50 |
| Coagulopathy | 24 | 28 | 28 | 24 | 26.00 |
| Cardiac Arrythmias | 23 | 26 | 27 | 29 | 26.25 |
| Weight Loss | 28 | 27 | 25 | 26 | 26.50 |
| Respiratory Device: Mild O_2_ Support | 26 | 29 | 29 | 27 | 27.75 |
| Pulmonary Circulation Disorders | 31 | 34 | 31 | 33 | 32.25 |
| Paralysis | 43 | 30 | 32 | 28 | 33.25 |
| Hypertension, Uncomplicated | 34 | 35 | 33 | 31 | 33.25 |
| Depression | 25 | 31 | 38 | 40 | 33.50 |
| Diabetes, Complicated | 32 | 33 | 36 | 36 | 34.25 |
| Peripheral Vascular Disorders | 33 | 32 | 30 | 46 | 35.25 |
| Race: Black | 35 | 39 | 42 | 32 | 37.00 |
| Hypertension, Complicated | 38 | 45 | 39 | 35 | 39.25 |
| Chronic Pulmonary Disease | 41 | 42 | 37 | 37 | 39.25 |
| Valvular Disease | 40 | 38 | 41 | 41 | 40.00 |
| Race: Other | 39 | 43 | 45 | 34 | 40.25 |
| Lymphoma | 51 | 36 | 34 | 42 | 40.75 |
| Deficiency Anemia | 36 | 37 | 46 | 47 | 41.50 |
| Renal Failure | 37 | 46 | 40 | 44 | 41.75 |
| Obesity | 47 | 41 | 35 | 45 | 42.00 |
| Sex | 42 | 40 | 49 | 38 | 42.25 |
| Alcohol Abuse | 44 | 50 | 50 | 39 | 45.75 |
| Hypothyroidism | 49 | 49 | 47 | 43 | 47.00 |
| Diabetes, Uncomplicated | 45 | 48 | 48 | 49 | 47.50 |
| Drug Abuse | 48 | 47 | 44 | 51 | 47.50 |
| Psychoses | 46 | 53 | 43 | 48 | 47.50 |
| Blood Loss Anemia | 52 | 44 | 51 | 54 | 50.25 |
| Rheumatoid Arthritis | 53 | 51 | 52 | 50 | 51.50 |
| Peptic Ulcer Disease | 50 | 52 | 53 | 52 | 51.75 |
| AIDS/HIV | 54 | 54 | 54 | 53 | 53.75 |
