# Supplementary material for: SafeNET: Initial development and validation of a real-time tool for predicting mortality risk at the time of hospital transfer to a higher level of care
Source: PLoS One. 2021 Feb 8;16(2):e0246669. doi: 10.1371/journal.pone.0246669 (PMC7870086; doi:10.1371/journal.pone.0246669)
Supplement: Attachment — Submitted filename: Response to Reviewers.docx [file pone.0246669.s004.docx]

*Wolff Center at UPMC*

4601 Baum Boulevard Suite 212

Pittsburgh, PA 15213

412-802-6560

December 11, 2020

Dear Dr. Ramesh Kumar,

We want to extend our gratitude to you for considering our manuscript entitled “SafeNET: Initial development and validation of a real-time tool for predicting mortality risk at the time of hospital transfer to a higher level of care”. We thank the reviewers for their time and appreciate their feedback. We have carefully considered the reviewers’ comments and have revised our manuscript accordingly. Changes from the original are marked by track changes in the version specified as such. As requested, an unmarked version is also available. Below is a point-by-point explanation describing how we addressed the concerns of the reviewers. We hope that with these changes will make the manuscript acceptable to PLOS ONE.

**Reviewer #1**
***The manuscript is well written with all the components addressed satisfactorily. The data has been analyzed and interpreted appropriately. English language used in the manuscript is good for grammar. The SafeNET algorithm used for coming up with a real-time tool to improve the good patient outcomes is an innovative idea.***

We are humbled by the positive assessment of Reviewer #1 and are thankful for their time and thoughtful comments regarding the SafeNET tool.

**Reviewer #2** 

***The manuscript titled "Development and validation of a real-time tool for predicting mortality risk at the time of hospital transfer", is a tremendous contribution of the authors towards an empirically deficient area of clinical practice. The contributions are understood to facilitate practitioners and clinicians in ascertaining the risk of mortality across specified time frames during hospital transfer of critically ill patients.***

We thank Reviewer #2 for their generous evaluation and for recognizing the clinical importance of the need for such a tool as SafeNET.

***It is suggested that the authors may slightly modify their background section to align more with the qSOFA and SafeNet tools, as currently it narrates about the existing tools but lesser mention of one of the prime tools that have been assessed in the study.***

We modified the arrangement and content of the second paragraph of the introduction to provide more clarity and detail about qSOFA and its current limitations. We also added a sentence to the last paragraph of the introduction to explicitly mention that we directly compared SafeNET with qSOFA as this is the most logical tool available for comparison.

***In methods section, the authors have narrated the adopted methodologies and approaches at length, and no major modifications are suggested. However, it would useful to have some more description about the process of independent variables selection from the existing tools and prioritization for clinical findings/observations.***

A thorough search of the literature identified 8 articles that described 7 applicable mortality risk tools. We reviewed these in depth to extract a comprehensive list of 70 independent variables that had been identified in previous research as promising predictors for this purpose. We then used information available to us through billing and electronic health records to identify which of these variables were readily accessible within a clinically relevant timeframe. This brought us down to 54 independent variables that were tested in our gradient boosting models. We added these additional details to the Independent and Dependent Variables portion of the Methods section.

***In the result section, it would be useful to relate the process of testing the tool with the clinical outcomes in the narrative as well. The tables and illustrations detail the statistical outputs well, however the narrative relates less with the 30 days and 90 days mortality aspects as observed in the study.***

We added in detailed results for the sensitivity, specificity, and positive predictive abilities for 30-day and 90-day outcomes. This information is in the last paragraph of the results section.

***There is reasonable attribution of study findings with other referenced materials in the discussion session, however, the conclusions mention about the deployment steps, rather than testing out the application of tools in other settings as well.***

We agree that the generalizability of SafeNET across different settings is unknown. We added a sentence to the last paragraph of the conclusion section to mention this limitation.

Thank you for your time and consideration.

Sincerely,

Stefanie Altieri Dunn
